# Endocytic pathway mediates refractoriness of insect *Bactrocera dorsalis* to RNA interference

**DOI:** 10.1038/srep08700

**Published:** 2015-03-03

**Authors:** Xiaoxue Li, Xiaolong Dong, Cong Zou, Hongyu Zhang

**Affiliations:** 1 State Key Laboratory of Agricultural Microbiology, Hubei Key Laboratory of Insect Resource Application and Sustainable Pest Control, Institute of Urban and Horticultural Entomology, College of Plant Science and Technology, Huazhong Agricultural University, Wuhan 430070, Hubei, People's Republic of China

## Abstract

RNA interference (RNAi) is a powerful and convenient tool for sequence-specific gene silencing, and it is triggered by double-stranded RNA (dsRNA). RNAi can be easily achieved in many eukaryotes by either injecting or feeding dsRNAs. This mechanism has demonstrated its potential in fundamental research on genetics, medicine and agriculture. However, the possibility that insects might develop refractoriness to RNAi remains unexplored. In this study, we report that the oriental fruit fly, *Bactrocera dorsalis*, became refractory to RNAi using orally administered dsRNA targeting endogenous genes. Furthermore, refractoriness to RNAi is not gene-specific, and its duration depends on the dsRNA concentration. RNAi blockage requires the endocytic pathway. Fluorescence microscopy indicated that in RNAi refractory flies, dsRNA uptake is blocked. Genes involved in the entry of dsRNAs into cells, including *chc, cog3, light* and others, are down-regulated in RNAi refractory flies. Increasing the endocytic capacity by improving F-actin polymerization disrupts RNAi refractoriness after both primary and secondary dsRNA exposures. Our results demonstrate that an insect can become refractory to RNAi by preventing the entry of dsRNA into its cells.

RNAi is a conserved mechanism by which endogenous genes are silenced by dsRNAs in a sequence-specific manner^1^. dsRNAs can be delivered into animals by various methods, including injection^2^, feeding^3^ and transgenic expression^4^. These dsRNAs are then processed by a member of the RNase III family, Dicer, into siRNAs of approximately 21 nucleotides in length^5^. The siRNA works as a guide and is loaded into the RNA-induced silencing complex (RISC), leading to sequence-specific mRNA cleavage^6^,^7^. RNAi can be cell-autonomous or non-cell-autonomous^8^. Cell-autonomous RNAi refers to RNAi that occurs within a single cell. Non-cell-autonomous RNAi refers to the ability of dsRNA to trigger RNAi in cells that are distant from the initial site of RNAi or the location where the dsRNA was introduced^8^. For the efficient use of RNAi in pest control, the focus must be on non-cell-autonomous RNAi caused by the feeding of dsRNAs^9^. The use of RNAi in pest management requires the dsRNA to be ingested in the lumen without being degraded, then taken up in the intestinal cells. Then, the dsRNA molecules can pass through the intestinal cells and into the body cavity, where they can act on other tissues, such as muscles^8^.

Because RNAi is easy to induce and highly efficient, it has been widely used throughout the scientific field. It has become a basic method in functional genetic studies. Genome-wide screens for genes involved in many biological pathways have been successfully carried out using RNAi-based methods^10^,^11^. Although RNAi-based therapy has not been fully realised, several achievements in related areas still provide hope for success^12^. RNAi is also a promising tool in agricultural science, and especially in pest management, as an environmentally friendly pesticide^13^.

RNAi experiments have been carried out in various insect orders, including the Diptera^14^, Coleoptera^15^, Lepidoptera^16^, Hemiptera^17^ and Isoptera^18^. Two landmark articles demonstrated the feasibility of the oral administration of dsRNA in insects^19^,^20^, supporting the use of RNAi in insect pest control. However, RNAi in insects has yielded varying results. Among the insects in which RNAi has been investigated, some appear to be RNAi-insensitive^21^. For example, feeding dsRNAs to adults of the Dipteran species *Drosophila melanogaster* failed to elicit RNAi^22^. In the Lepidopteran species *Spodoptera litura*, gene silencing using dsRNA was observed only with injection; the feeding method failed^23^. An attempt to silence the *nitroporin 2* gene in 4th instar larvae of *Rhodnius prolixus* failed despite the large quantity of dsRNA that was used (80 μg)^17^.

It has been demonstrated that both dsRNAs and siRNAs activate the type I interferon (IFN) system in mammalian cells^24^. Unlike in vertebrates, it is generally believed that IFN responses do not exist in invertebrates because the critical genes or major effectors of the IFN pathway are absent in these species^25^. Recent studies have shown that the RNAi pathway plays an important role in invertebrate viral immunity^26^. Robalino et al. (2004) injected dsRNAs derived from vertebrate immunoglobulin genes, fish non-coding genomic DNA, bacterial vector sequences, and the Taura syndrome virus into marine shrimp. Each of these sequences induced protection against infection with the white spot syndrome virus (WSSV)^25^. Administration of dsRNA that targets either virus-specific or non-specific sequences can trigger an antiviral response that controls viral infections in honey bees^27^. However, importantly, this viral immunity was not sequence-specific; it could be activated by dsRNAs derived from any sequence^25^.

To date, there is little, if any, evidence indicating that an organism can become refractory to dsRNA-induced RNAi. Here, we report that the insect *B. dorsalis* can become refractory to RNAi triggered by feeding. Refractoriness is caused by a decrease in the endocytic entry of dsRNA into the intestinal cells, thus preventing RNAi. Digital gene expression (DGE) and qPCR analysis show that several genes involved in endocytosis are down-regulated. Increasing the endocytic capacity by promoting actin assembly can reverse the refractoriness. The mechanism of RNAi refractoriness uncovered here might explain why RNAi is difficult to achieve in certain insects.

## Results and Discussion

### Feeding of dsRNAs targeting endogenous genes induces protection against secondary RNAi

**Figure 1:**
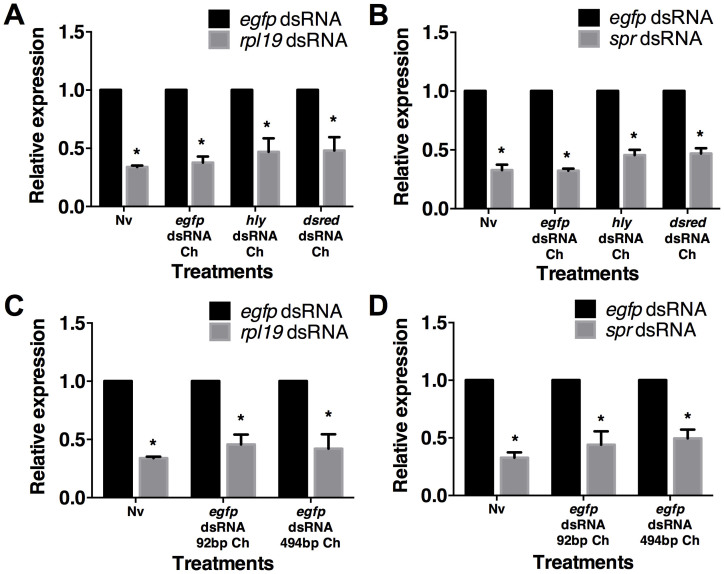
Feeding dsRNA that targets exogenous genes do not affect subsequent RNAi. (A) The effect of dsRNA that targets exogenous genes on a second exposure to *rpl19* dsRNA. (B) The effect of dsRNA that targets exogenous genes on a second exposure to *spr* dsRNA. (C) The effect of 92 bp and 494 bp *egfp* dsRNA on a second exposure to *rpl19* dsRNA. (D) The effect of 92 bp and 494 bp *egfp* dsRNA on a second exposure to *spr* dsRNA. Normalised target gene expression is reported relative to the expression after *egfp* dsRNA treatment, which was set to 1. All error bars represent the SE of the mean of three independent biological replicates. * indicates a statistically significant difference in *spr* or *rpl19* expression between *rpl19* dsRNA or *spr* dsRNA and the control *egfp* dsRNA treatments (*P* < 0.01).

Unlike vertebrates, invertebrates lack acquired immunity^28^. However, growing empirical evidence suggests that previous exposure to a parasite can lead to increased protection in response to a subsequent challenge^29^. In invertebrates, this phenomenon is termed “immune priming”^29^. In this study, we aimed to determine if primary RNAi could further influence secondary RNAi in *B. dorsalis*. First, we tested the effects of prior RNAi using a dsRNA sequence targeting a non-endogenous gene on a subsequent RNAi by exposure of dsRNA targeting an endogenous gene. These dsRNAs were derived from enhanced green fluorescent protein (*egfp*) (186 bp), *Discosoma* sp. red fluorescent protein (*dsred*) (192 bp), the *hly* gene from *Listeria monocytogenes (hly)* (207 bp). The flies were divided into two groups. The challenged group (Ch) was fed an artificial diet containing one of the above dsRNAs for 6 hr. The other group, referred to as the naive group (Nv), was fed a normal artificial diet. Five days post-first exposure (dpe), both the challenged group and the naive group were orally administered a dsRNA targeting either the *sex peptide receptor (spr)* gene (280 bp) or the *ribosomal protein L19 (rpl19)* gene (205 bp). *rpl19* is housekeeping genes encoding ribosomal protein L19 subunit. Our results showed that *rpl19* is uniformly expressed in all tissues in *B. dorsalis* (Figure S1). *spr* mediates post-mating behaviour changes in female flies. *spr* is highly expressed in the midgut, and is also expressed in the head and the reproductive organs in *B. dorsalis* (data unpublished). The results showed that, in the first round of RNAi, none of the three exogenous dsRNAs affected the expression of *spr* or *rpl19* (Figure S2A, B). In addition, *spr* dsRNA and *rpl19* dsRNA did not have mutual effect on each other expression (Figure S2A, B). In the secondary RNAi assay, both the naive group and the challenged group exhibited strong RNAi; the expression of the target genes was reduced by more than 60%, demonstrating the efficiency of the RNAi (Figure 1A, B). These results suggest that exposure to a dsRNA targeting a non-endogenous gene did not reduce the effect of subsequent exposure to a dsRNA against an endogenous gene. We also tested if dsRNA length could affect the secondary RNAi outcome. The results showed that exposure to 92 bp and 494 bp *egfp* dsRNAs did not change the effect of a secondary RNAi targeting either *rpl19* or *spr*. Both the naïve and the challenged group exhibited strong RNAi (Figure 1C, D). It indicates that the length of dsRNAs targeting exogenous genes do not affect the outcome.

**Figure 2:**
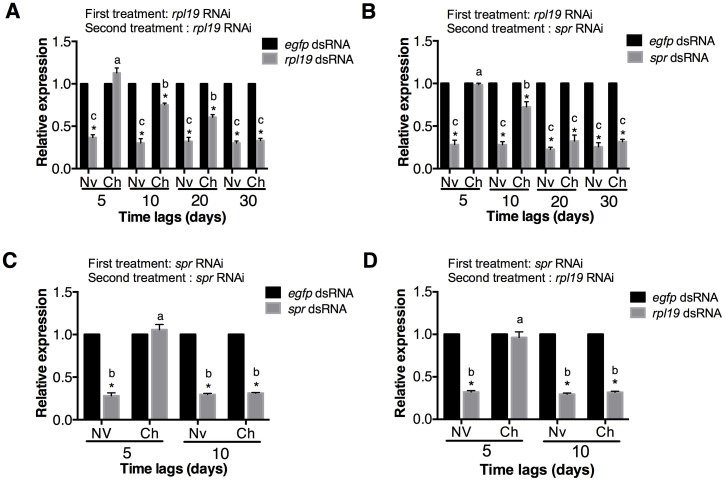
Feeding dsRNA that targets endogenous genes induces protection against subsequent RNAi. (A) RNAi refractoriness to secondary *rpl19* dsRNA exposure, primed by *rpl19* dsRNA. (B) RNAi refractoriness to secondary *spr* dsRNA exposure, primed by *rpl19* dsRNA. (C) RNAi refractoriness to secondary *spr* dsRNA exposure, primed by *spr* dsRNA. (D) RNAi refractoriness to secondary *rpl19* dsRNA exposure, primed by *spr* dsRNA. Normalised target gene expression is reported relative to the expression after *egfp* dsRNA treatment, which was set to 1. All error bars represent the SE of the mean of three independent biological replicates. * indicates a statistically significant difference in *spr* or *rpl19* expression between *rpl19* dsRNA or *spr* dsRNA and the control *egfp* dsRNA treatments (*P* < 0.01). Different letters indicate a significant difference in *rpl19* or *spr* expression among the *rpl19* dsRNA or *spr* dsRNA treatments (*P* < 0.01).

Next, we used *rpl19* dsRNA as the first exposure for the challenged group, whereas *egfp* dsRNA was used for the naive group. *rpl19* expression decreased by 65.2% after the first exposure (Figure S3A). The RNAi effect disappeared four days after the first RNAi (Figure S3A). As expected, the naive group showed efficient RNAi after secondary exposure to a dsRNA targeting *rpl19*; in this group, *rpl19* expression decreased by 66% at 5 dpe (Figure 2A). However, in the challenged group, after secondary RNAi, depletion of *rpl19* could not be observed at 5 dpe. This phenomenon suggests that an initial exposure to *rpl19* dsRNA prevents RNAi-induced gene silencing after a second exposure to the same dsRNA.

We next examined the duration of RNAi refractoriness. We set the time lag between the two RNAi exposures to 10, 20 or 30 days (Figure S4). First we examined *rpl19* and *spr* gene expression in the untreated flies. Our results suggest that there is no expression level fluctuation for *spr* and *rpl19* during 30 days in the untreated flies (Figure S5A, B). qPCR results of secondary RNAi suggested that, although RNAi refractoriness decreased with time, it was still observable after secondary RNAi at 10 and 20 dpe. After a secondary oral administration of *rpl19* dsRNA, *rpl19* mRNA levels were only down-regulated by 25% and 39% at 10 and 20 dpe, respectively, in the challenged group (Figure 2A). By contrast, insects that were first treated with either *egfp* dsRNA or *rpl19* dsRNA exhibited strong *rpl19* depletion after 30 dpe, indicating that the RNAi refractory period lasted no more than 30 days after initial exposure (Figure 2A). These results demonstrated that exposure to orally administered *rpl19* dsRNA induced a change in the insect host that made it refractory to the effects of subsequent RNAi targeting *rpl19*. Our results show that feeding *B. dorsalis* a dsRNA against a specific gene reduced the RNAi efficiency of a second exposure to dsRNA against the same gene.

### RNAi refractoriness is not sequence-specific and is influenced by the concentration of the priming dsRNA

We next examined if RNAi refractoriness primed by *rpl19* dsRNA was also effective against secondary RNAi targeting *spr* at 5 dpe. The results showed that prior ingestion of *rpl19* dsRNA provided protection against subsequent RNAi targeting *spr*. Feeding the naive group with *spr* dsRNA lead to an approximately 70% down-regulation of *spr* expression after secondary RNAi (Figure 2B). By contrast, the group initially challenged with *rpl19* dsRNA showed no reduction in *spr* gene expression after secondary *spr* RNAi at 5 dpe (Figure 2B). There was no significant difference between the naive group and the challenged group when secondary *spr* gene silencing was assessed at 20 dpe (Figure 2B). We then tested if a dsRNA targeting a gene other than *rpl19* could elicit RNAi refractoriness. To address this question, we chose *spr* as a target gene for initial dsRNA exposure. The results showed that, in the challenged group, the initial exposure to *spr* dsRNA induced refractoriness towards to secondary exposure to both *spr* dsRNA and *rpl19* dsRNA (Figure 2C, D). The refractory state primed by *spr* dsRNA protected *B. dorsalis* for 5 dpe after secondary exposure to both *spr* and *rpl19* dsRNA. However, this refractory period was much shorter than that provoked by *rpl19* dsRNA. There was no difference in gene expression between the challenged and the naive groups fed either *rpl19* dsRNA or *spr* dsRNA after 10 dpe (Figure 2C, D). These results indicate that a refractory state can be provoked by dsRNAs with different sequences. Because we did not observe this refractory state using *egfp* or other dsRNAs targeting exogenous genes, we assume that only dsRNAs targeting endogenous genes can trigger RNAi refractoriness.

**Figure 3:**
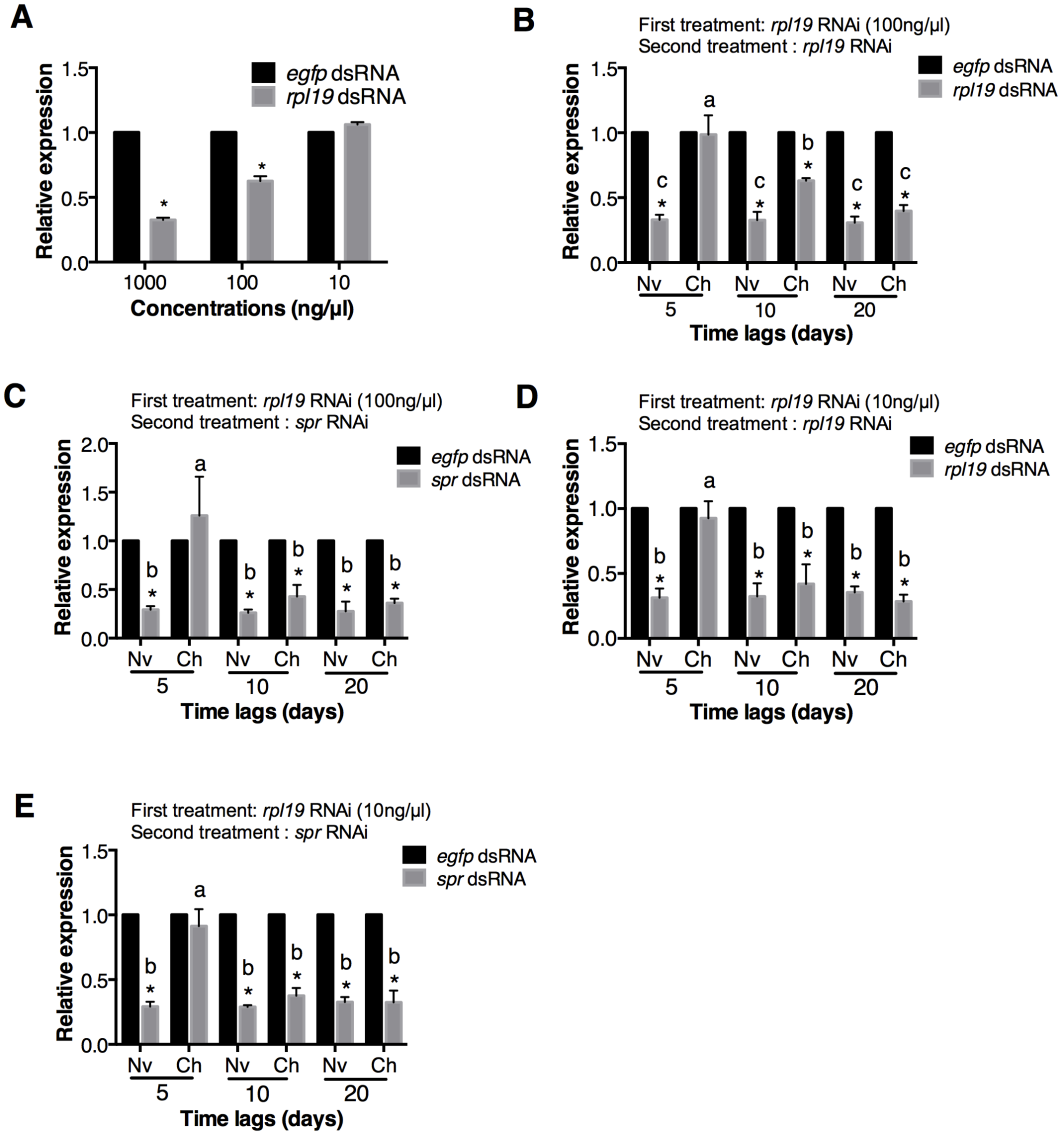
RNAi refractoriness primed by different concentrations of *rpl19* dsRNA. (A) The RNAi effect induced by oral administration of different concentrations of *rpl19* dsRNA. (B) RNAi refractoriness to secondary *rpl19* dsRNA exposure, primed by 100 ng/μl *rpl19* dsRNA. (C) RNAi refractoriness to secondary *spr* dsRNA exposure, primed by 100 ng/μl *rpl19* dsRNA. (D) RNAi refractoriness to secondary *rpl19* dsRNA exposure, primed by 10 ng/μl *rpl19* dsRNA. (E) RNAi refractoriness to secondary *spr* dsRNA exposure, primed by 10 ng/μl *rpl19* dsRNA. Normalised target gene expression is reported relative to expression in the *egfp* dsRNA control, which was set to 1. All error bars represent the SE of the mean of three independent biological replicates. * indicates a statistically significant difference in *spr* or *rpl19* expression between *rpl19* dsRNA or *spr* dsRNA and the control *egfp* dsRNA treatments (*P* < 0.01). Different letters indicate a significant difference in *rpl19* or *spr* expression among the *rpl19* dsRNA or *spr* dsRNA treatments (*P* < 0.01).

We next explored if the concentration of dsRNA used in the first exposure influenced RNAi refractoriness. The results showed that 100 ng/μl *rpl19* dsRNA decreased *rpl19* expression by 38%, but that 10 ng/μl *rpl19* dsRNA did not decrease *rpl19* expression (Figure 3A). Both dsRNA concentrations could block RNAi-mediated gene silencing after secondary exposure to two different dsRNAs 5 dpe. Nevertheless, the refractory periods were shorter than those induced by the 1000 ng/μl treatment. In the group treated with 100 ng/μl, secondary exposure 10 dpe decreased *rpl19* expression by 40% in the challenged group and by 70% in the naive group (Figure 3B). No difference could be observed between the naive and challenged groups after secondary exposure 20 dpe (Figure 3B). This shortened period of refractoriness was more obvious when analysing its effect on a second exposure to *spr* dsRNA; in this case, the RNAi refractory period lasted no longer than 10 days (Figure 3C). The refractoriness induced by the 10 ng/μl *rpl19* dsRNA were even shorter than those induced by 100 ng/μl *rpl19* dsRNA (Figure 3D, E). These results clearly illustrated that the duration of RNAi refractoriness to dsRNAs is correlated with the dsRNA concentration used for priming. In addition, we show that effective target gene silencing after the first exposure was not a prerequisite for RNAi refractoriness. Since that only the dsRNAs targeting endogenous genes could induce the RNAi refractoriness, this process might involves the dsRNA and target mRNA interaction. In addition, the fact that target gene silencing is not necessary for the RNAi refractoriness demonstrated that low level of dsRNA and target mRNA interaction, although not enough to silence target genes, is enough to elicit RNAi refractoriness. Thus, the high level of this interaction caused by high concentration of dsRNA guarantees a prolonged refractoriness.

### Disruption of the endocytic pathway inhibits dsRNA entry

**Figure 4:**
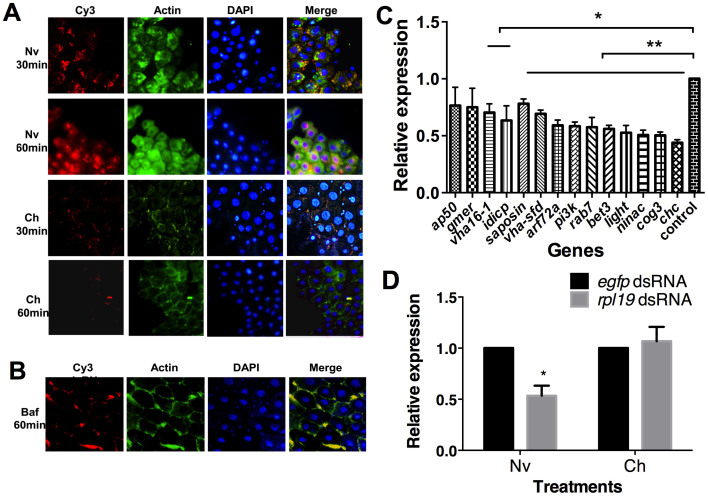
The cellular entry of dsRNA in *rpl19* dsRNA-challenged flies is disrupted. (A) Subcellular localization of Cy3-labelled dsRNA in the *egfp* dsRNA-treated naive group and the *rpl19* dsRNA-treated challenged group over time. (B) Subcellular localization of Cy3-labelled dsRNA after incubation in the presence of Baf. (C) Expression levels of genes required for the endocytic entry of dsRNAs. (D) Microinjection of *rpl19* dsRNA in the second RNAi. *, *P* < 0.05, and **, *P* < 0.01. Normalised expression of the target genes is given relative to their expression in the *egfp* dsRNA-treated control, which was set to 1. All error bars represent the SE of the mean of three independent biological replicates.

We next investigated the molecular mechanisms underlying RNAi insensitivity. In our conditions, the midgut is expected to be involved in dsRNA uptake^13^. Cy3-labelled dsRNAs were used to track dsRNA in the midgut cells of naive and challenged insects. Fluorescence microscopy revealed differences between the *rpl19* dsRNA-challenged group and the *egfp* dsRNA naive group after secondary exposure to *rpl19* dsRNA (Figure 4A). In the naive group, dsRNA begins to enter the cell after 30 minutes of incubation, and after another 30 minutes, the dsRNAs accumulate in a spot near the nucleus. This indicates that *egfp* dsRNA treatment did not impair the ability of dsRNAs from the second exposure to enter the midgut cells. However, in the *rpl19* dsRNA-challenged group, the dsRNAs failed to enter the midgut cells, as indicated by labelled dsRNAs accumulating outside the cells after both 30 and 60 min. These fluorescence microscopy observations demonstrate that the challenged group failed to respond to dsRNA feeding due to impaired dsRNA cellular uptake.

Two mechanisms of dsRNA uptake have been identified: one is mediated by the SID-1 transmembrane protein, and the other is mediated by endocytosis^30^. However, there is no *sid-1* gene in several insect genomes, including that of *D. melanogaster*^9^. Two independent works have shown that *D. melanogaster* relies on receptor-mediated endocytosis to take up dsRNA^31^,^32^. Bafilomycin A1 (Baf), a specific inhibitor of vacuolar proton ATPases, is often employed to demonstrate the requirement for low endosomal pH. Our results showed that pretreating the *B. dorsalis* midgut with baf blocked dsRNA entry into the midgut cells (Figure 4B). This strongly supports the notion that, in *B. dorsalis*, the entry of dsRNA into the cells requires endocytosis. We next examined the expression of genes reported to be responsible for the cellular entry of dsRNA in *D. melanogaster*^31^. These genes, such as *chc, rab7, light* and *saposin*, influence several crucial steps of endocytosis, including vesicle formation and transport, intracellular transport and lipid metabolism. Saleh et al. have shown that, in cell culture assays, RNAi against each of these genes inhibits dsRNA entry^31^. qPCR results showed that, in the *rpl19* dsRNA-challenged group, most of these genes were repressed 24 hr after secondary exposure (Figure 4C). For example, *chc*, a key gene required for clathrin-mediated endocytosis that encodes the clathrin heavy chain protein, was down-regulated by more than 60%. A similar level of down-regulation was observed for the *rab7, arf72a, light* (a vacuolar protein sorting *Vsp41* orthologue) and *vacuolar H^+^-ATPase (V-H-ATPase)* genes, which encode components of the endocytic vesicle trafficking and protein sorting pathways. Other genes, including members of the Golgi complex (COG) family (e.g., *ldlCp, cog3* and *bet3*) were also down-regulated. *nina c*, which is required for actin polymerization and cytoskeletal organization, was also down-regulated after the second exposure. These results raised the hypothesis that RNAi refractoriness is caused by a decrease in endocytosis. In order to find if the RNAi refractoriness is systemic, we directly injected *rpl19* dsRNA into the flies to see if it could bypass RNAi refractoriness. The result showed that, in the challenged group, direct injection of dsRNA could not reduce the expression of the target gene at 5 dpe. This result demonstrated that this refractoriness is systemic (Figure 4D).

**Figure 5:**
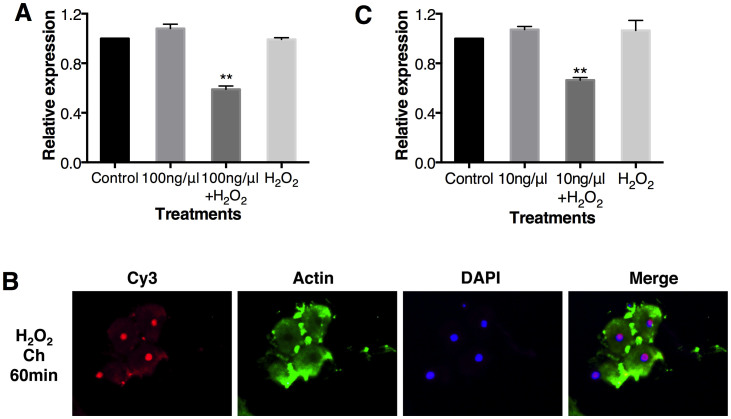
H_2_O_2_ can disrupt RNAi refractoriness. (A) Co-feeding of 100 ng/μl *rpl19* dsRNA with H_2_O_2_ in the second exposure. (B) Subcellular localization of Cy3-labelled dsRNA in the flies co-incubated with 5% H_2_O_2_ and *rpl19* dsRNA in challenged group. (C) Co-feeding of 10 ng/μl *rpl19* dsRNA with H_2_O_2_ in the first exposure. ** (*P* < 0.01) indicate significant differences in gene expression between the *rpl19* dsRNA treatment and the control. The normalised expression of the target gene is given relative to its expression in the *egfp* dsRNA control, which was set to 1. All error bars represent the SE of the mean of three independent biological replicates.

Actin assembly has been shown to be an essential element of endocytosis^33^. Hydrogen peroxide (H_2_O_2_) induces the formation of cellular F-actin in a dose-dependent manner^34^. To test the role of endocytosis in RNAi refractoriness, we investigated the ability of H_2_O_2_ to disrupt RNAi refractoriness. Applying H_2_O_2_ during the secondary exposure disrupted RNAi refractoriness; a second exposure with 100 ng/μl *rpl19* dsRNA and 5% H_2_O_2 _resulted in a 41.1% decrease in target gene expression compared with the *egfp* dsRNA treatment (Figure 5A). This is also supported by fluorescence microscopy; we found that dsRNA successfully entered the cells after co-incubation with H_2_O_2_ (Figure 5B). Because increasing the endocytic capacity with H_2_O_2_ led to a higher level of RNAi-mediated gene silencing, our results strongly suggest that refractoriness to RNAi is mediated by a decrease in endocytosis.

### DGE analysis reveals that RNAi refractoriness is intrinsic

Our results showed that 10 ng/μl *rpl19* dsRNA could not efficiently reduce *rpl19* expression (Figure 3A). Therefore, we tried to determine if RNAi insensitivity could also occur during the first dsRNA exposure. RNAseq analysis was used to identify genes that were differentially expressed after the first dsRNA exposure. RNA samples extracted at 8, 12 and 24 hr after exposure to 10 ng/μl *rpl19* dsRNA or *egfp* dsRNA were mixed together and sequenced.

A total of 5,973,846 and 6,512,317 raw paired-end reads with a length of 100 bp, corresponding to 10 ng/μl *rpl19* dsRNA treatment and 10 ng/μl *egfp* dsRNA treatment libraries were generated respectively. A total of 5,793,444 and 6,363,092 clean reads were left after removing reads with adaptors, reads containing poly N and low quality reads from raw data (Table S1). We mapped the sequences of two DGE libraries to the reference transcriptome dataset of *B. dorsalis*^35^, which contains 48,876 unigenes (Figure S6). This analysis identified 190 genes whose expression varied in response to feeding *rpl19* dsRNA compared with *egfp* dsRNA treatment. The gene set comprised 26 up-regulated and 164 down-regulated genes. Gene Ontology (GO) enrichment analysis indicated that the genes were enriched for 5 biological processes (p < 0.001), including translation regulator activity, cell motility and transport (Table S2). KEGG analysis of the differentially expressed genes showed that they function in several processes (Figure S7). The up-regulated genes mainly function in metabolism and translation, whereas the down-regulated genes primarily function in metabolism, translation and transcription, folding, sorting and degradation and transport and catabolism.

Importantly, several genes that play a crucial role in endocytosis were found to be down-regulated in the 10 ng/μl *rpl19* dsRNA-treated flies (Table 1). *chc* and *hsc70*, which encode factors that work with auxin to uncoat CCV from cargo^36^, as well as *saposin*, were found to be down-regulated. In addition, the expression of three genes encoding different F-actin isoforms, *actin 3, actin 4* and *actin 5*, was reduced. The dynamic polymerization of actin has a central role in clathrin-mediated endocytosis, which reshapes the plasma membrane^37^. The expression of three kinases, *hexo* *kinase 2*, *map2k1* and *pgk1*, which regulate endocytosis^38^, was also repressed. However, we failed to identify genes that had been previously characterised as involved in insect immunity; these genes encode components of the Toll, Imd and Jak/STAT pathways. This is consistent with the findings of Flenniken and Andino^27^, who used microarrays to identify genes involved in the dsRNA-mediated antiviral response in honey bees; they did not identify any classical immunity genes. These results, as well as ours, suggest that dsRNA-mediated antiviral defence may involve unique genes and signal transduction cascades^27^. In addition, we did not find core genes in the RNAi machinery, like *dicer 2* and *argonaute 2*, in the DGE analysis, indicating the RNAi refractoriness is not due to the different activity of the RNAi machinery. The RNAseq results imply that endocytosis-mediated RNAi refratoriness also occurs at the first dsRNA exposure. To validate this hypothesis, we co-fed flies with 5% H_2_O_2_ and 10 ng/μl *rpl19* dsRNA, a dsRNA concentration that does not reduce *rpl19* expression. However, *rpl19* expression still decreased 33.5% relative to the *egfp* dsRNA treatment (Figure 5C). This result demonstrates that H_2_O_2_, which influences endocytosis, can also influence RNAi-induced gene silencing. This observation reinforces our conclusion that variation in endocytic capacity can influence RNAi and that RNAi insensitivity is linked to a decrease in endocytic activity.

## Conclusions

Taken together, our findings indicate that *B. dorsalis* possesses a mechanism to down-regulate dsRNA-mediated RNAi. We also demonstrated that primed RNAi refractoriness involves clathrin-mediated endocytosis. We hypothesize that our findings will extend to other insect species. In line with this assumption, other works have already shown that receptor-mediated endocytosis influenced dsRNA entry in *D. melanogaster*^31^. Our conclusion is also supported by the work of Whyard^22^, who showed that feeding dsRNA encapsulated by transfection reagents could induce RNAi in four different *Drosophila* species, whereas direct dsRNA feeding did not work. This suggests that a mechanism involved in dsRNA entry is impaired in *Drosophila* species. We speculate that this defect is linked to a reduction in endocytosis. A recent report focused on the early response of *Drosophila S2* cells to viruses^39^. Interestingly, this work showed that receptors such as Sr-CI, Eater and TepI were significantly down-regulated after pathogenic virus treatments, and significant changes in phagocytic activity were observed. It is known that together, Sr-CI and Eater contribute to more than 90% of dsRNA uptake into S2 cells. Considering the evolutionary conservation and functional relevance of the dsRNA entry pathway in intact organisms^31^, this change in membrane transport capacity might be common in host-pathogen interactions.

RNAi is an important viral defence mechanism in insects. The mechanism that we uncovered might affect the viral defences of insects and could explain the variability observed between the various physiological states that influence endocytosis. It also suggests that infection by a virus could influence RNAi silencing against a second virus or against a second exposure to the first virus. Furthermore, our work also has important consequences for the use of RNAi in other insects; it could explain why RNAi is difficult to achieve in some insect species. It also provides a solution to this problem by showing that promoting endocytosis enhances RNAi. In summary, our findings provide a perspective into mechanisms that allow invertebrates to protect their genetic information from non-self dsRNAs.

## Methods

### Fly rearing

*B. dorsalis* flies were reared at the Institute of Urban and Horticultural Pests at Huazhong Agricultural University. Adult flies were maintained in cages at 28°C under a 12 hr light:12 hr dark photoperiod and fed an artificial diet consisting of 2.5% yeast extract, 7.5% sugar, 2.5% honey, 0.4% agar and 87% H_2_O.

### Plasmid construction

Total RNA was extracted from adult flies using RNAiso Plus reagent (TaKaRa, Japan). First strand cDNA was synthesised using a PrimeScript 1st Strand cDNA Synthesis Kit (TaKaRa, Japan). PCR fragments from each gene were cloned into the SacI and HindIII sites of the L4440 plasmid. The *egfp* and *dsred* fragment were cloned from the PUbnlsEGFP plasmid and the PUbnlsRED palsmid. All the primers used in the experiments were listed in Table S3. All the HT115 (DE3) competent cells lacking RNase III were prepared using standard CaCl_2_ methodology and were transformed with recombinant plasmid DNA.

### dsRNA preparation and quantification

A single HT115 (DE3) colony was cultured overnight in LB at 37°C with shaking at 220 rpm. The culture was diluted 100-fold in 800 mL 2 × YT supplemented with 75 μg/mL ampicillin and 12.5 μg/mL tetracycline and cultured at 37°C to an OD600 of 0.5. Production of T7 polymerase was induced with 0.4 mM IPTG, and the bacteria were incubated with shaking for an additional 4 hr at 37°C. Total nucleic acids were extracted^40^. The bacterial pellets were resuspended in 1 M ammonium acetate/10 mM EDTA, and an additional volume of phenol:chloroform:isoamyl alcohol (25:24:1) was added. The samples were incubated at 65°C for 30 min and centrifuged at 12,000 g for 15 min. The upper phase was mixed with isopropanol, incubated at −20°C overnight and centrifuged at 12,000 g for 30 min. The nucleic acid pellet was resuspended in TE. For dsRNA quantification, the nucleic acids were treated with RQ1 RNase-free DNase (Promega, USA) and RNase A solution (Promega, USA). The concentration was determined using a NanoDrop 1000 (Thermo, USA). The dsRNAs were loaded onto a 2% agarose gel, stained with ethidium bromide, and photographed to determine integrity.

### Microinjection

Needles were prepared with a puller at 60°C (PC-10, Narishige, Tokyo Japan). Microinjection was performed using an Eppendorf micromanipulation system. The injection condition was set to a Pi of 300 hpa and a Ti of 0.3 s. A total of 100 ng *egfp* dsRNA or *rpl19* dsRNA was injected into the flies in the challenged group.

### Bioassays

Flies were collected five to ten days after emergence. For each treatment, flies were dehydrated/starved for 24 hr. The artificial diet was cut into circular pieces with 6 cm diameters. Each piece was covered with 400 μl of dsRNA solution prepared as described above. Unless mentioned, the concentration of dsRNA is 1000 ng/μl in the feeding bioassay. The flies were fed the artificial diet supplemented with dsRNA starting at 8:00 am and were returned to a normal artificial diet at 14:00 pm the same day. The experiments were performed in triplicate. For immune priming experiments, the flies were divided into two groups. For the first RNAi exposure, one group was fed *rpl19* dsRNA or *spr* dsRNA as described above. These flies were referred to as the challenged group. The other group, referred to as the naïve group, was fed *egfp* dsRNA. We set different lag times between the first and second dsRNA exposures (5, 10, 20 and 30 days). At each time point, we collected flies from both the challenged and naïve groups and examined their responses to a second dsRNA exposure (either *rpl19* dsRNA or *spr* dsRNA). In this experiment, *egfp* dsRNA was used as the control to determine the RNAi effect. In all cases, samples were collected 24 hr after the second exposure. All experiments were repeated in triplicate. For the RNAi refractoriness disruption experiments, 5% H_2_O_2_ was co-fed with the dsRNA.

### Real-time PCR

For each treatment, 10 flies were collected for RNA extraction. RNA was extracted using RNAiso Plus reagent (TaKaRa, Japan). cDNA was synthesised from 100 ng total RNA using Transcript RT Master Mix (TaKaRa, Japan) according to the manufacturer's instructions. Real-time RT-PCR was performed using BioRad SYBR Green qPCR mix (BioRad, USA) according to the manufacturer's instructions, on a BioRad MyIQ2 machine. All RNA samples were analysed in triplicate. The reactions included 2 μl cDNA, 10 μl SYBR Green mix (BioRad, USA), 0.8 μl each of forward and reverse primers and 6.4 μl ddH_2_O. The reactions were set up in 96-well real-time PCR plates (BioRad, USA). The thermocycler conditions were 95°C for 30 s, followed by 40 cycles at 95°C for 15 s and 60°C for 30 s. Melting curve analysis was performed at the end of each amplification run to confirm the presence of a single peak. The thermocycler conditions for the melting curve analysis were 55°C for 60 s, followed by 81 cycles starting at 55°C for 10 s with a 0.5°C increase each cycle. To avoid off-target effects, qPCR primers were designed to detect the parts of the transcript outside the dsRNA target. *sdha* was used as a reference gene, and it was selected from a pool of candidate genes using geNorm. The data were analysed using the 2^−ΔΔ*C*^_T_ method^41^ using IQ5 standard edition ver. 2.1 (BioRad, USA). The expression of *rpl19* and *spr* was quantified relative to the levels of *rpl19* and *spr* in the flies fed *egfp* dsRNA. Biological experiments were performed independently and in triplicate. All results from experimental replicates were analysed using Student's t-test or a one-way analysis of variance (ANOVA) and a Duncan's test using SPSS 20 (IBM Corporation, USA).

### DGE analysis

A mixed sample of flies was collected 8, 12 and 24 hr after exposure to 10 ng/μl dsRNA. Total RNA was extracted and digested with DNase I (Ambion, USA). mRNA was purified with a Micropoly(A)Purist™ mRNA purification kit(Ambion, USA)following the manual's instructions. cDNA synthesis was performed with a SuperScript Double-stranded cDNA Synthesis Kit (Invitrogen, USA). cDNA was purified using Ampure beads (Agencourt, USA). The purified cDNA was used to prepare a library using a TruSeq™ DNA sample Prep Kit-Set A (Illumina, USA), and PCR amplification was performed using a TruSeq PE Cluster Kit (Illumina, USA). Finally, the products were sequenced on an Illumina HiSeqTM 2000 System (Illumina, USA) and 100 bp pair-end reads were generated. Clean reads were mapped to a *B. dorsalis* transcriptome dataset^35^. Expression values were calculated in units of RPKM. Statistical analysis to identify differentially expressed genes was performed using an MA plot-based method with a random sampling model in DEGseq^42^.

### Immunofluoresence microscopy

dsRNA was fluorescently labelled using a Silencer siRNA Labelling Kit with Cy3 (Ambion, USA). Labelling of dsRNA was verified by decreased electrophoretic mobility compared with unlabelled dsRNA on an agarose gel. *B. dorsalis* midgut tissue from both the challenged and naïve groups were incubated with labelled dsRNA. For bafilomycin A1 treatment, midgut tissue was first incubated with 0.2 μM Baf for 30 minutes. Cy3-labelled dsRNA was then added to the reaction. The tissue was fixed for 20 min in 4% formaldehyde. Actin was visualized with Acti-stain™ 488 fluorescent phalloidin (Cytoskeleton, Inc., USA) following the instruction manual. Nuclei were counterstained with DAPI. Images were captured on an Olympus IX71 microscope driven by cellSens Dimension software (Olympus, Japan). All images were imported into and processed in Adobe Photoshop (Adobe, USA).

## Author Contributions

H.Z. and X.L. conceived the study and participated in its design. H.Z. provided the materials for the study. X.L. and X.D. performed the bioassays and DGE analysis. X.L. and C.Z. performed the immunofluorescence microscopy and H_2_O_2_ bioassays. H.Z. and X.L. performed the data analysis. H.Z. and X.L. wrote the manuscript. All authors read and approved the final manuscript.

## Supplementary Material

Supplementary Information Supplementary information

## Figures and Tables

**Table 1:** List of representative differentially expressed genes found by DGE

Function	Gene	Fold change	q-value
Endocytosis	*cdc42*	0.32	9.99E − 03
	*chc*	0.47	5.60E − 07
	*hsc70*	0.32	1.29E − 12
	*ehd1*	0.62	1.20E − 02
Peroxisome	*saposin*	0.42	1.82E − 10
Phagosome	*vha16-1*	−0.20	2.55E − 02
	*actin 3*	0.38	6.19E − 03
	*actin 5*	1.01	1.09E − 16
	*actin 4*	0.21	1.44E − 02
Insulin signalling	*hexokinase2*	0.40	2.28E − 08
	*map2k1*	0.41	1.10E − 02
Glycolysis	*pgk1*	0.46	9.63E − 06
