# Supplementary material for: Endocytic pathway mediates refractoriness of insect Bactrocera dorsalis to RNA interference
Source: Sci Rep. 2015 Mar 3;5:8700. doi: 10.1038/srep08700 (PMC4346973; doi:10.1038/srep08700)
Supplement: Supplementary Information [file srep08700-s1.doc]

**Supplementary Information**

Endocytic pathway mediates refractoriness of insect Bactrocera dorsalis to RNA interference

Xiaoxue Li^1^ Xiaolong Dong^1^ Cong Zou^1^ Hongyu Zhang^1*^

^1^State Key Laboratory of Agricultural Microbiology, Hubei Key Laboratory of Insect Resource Application and Sustainable Pest Control, Institute of Urban and Horticultural Entomology, College of Plant Science and Technology, Huazhong Agricultural University, Wuhan 430070, Hubei, People’s Republic of China

*Corresponding author Hongyu Zhang: [hongyu.zhang@mail.hzau.edu.cn](mailto:hongyu.zhang@mail.hzau.edu.cn)

**
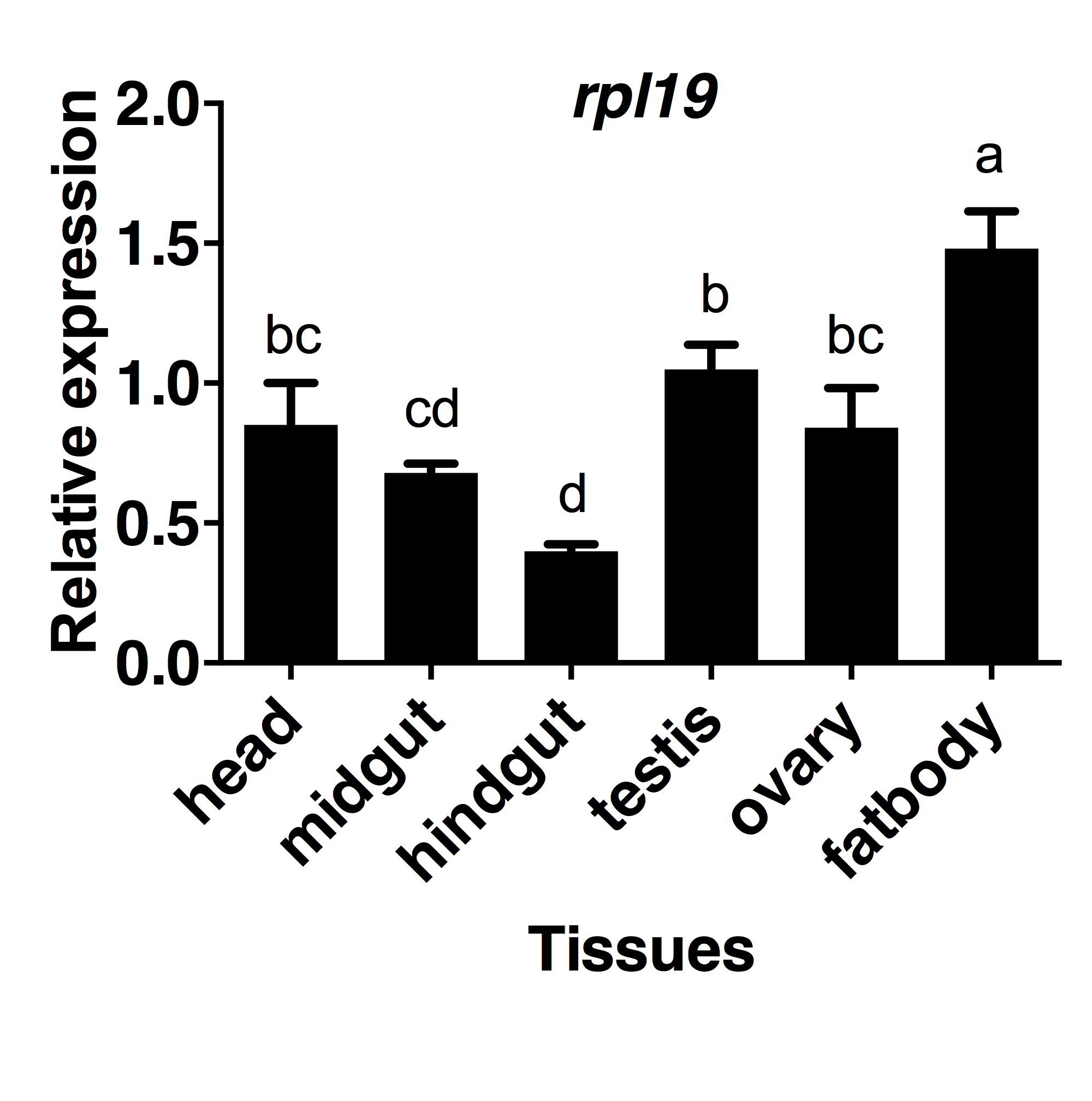
**

**Figure S1 The expression pattern of *rpl19* in *B. dorsalis*.** All error bars represent the SE of the mean of three independent biological replicates. Different letters indicate a significant difference in *rpl19* expression among different tissues (*P* <0.01).

**
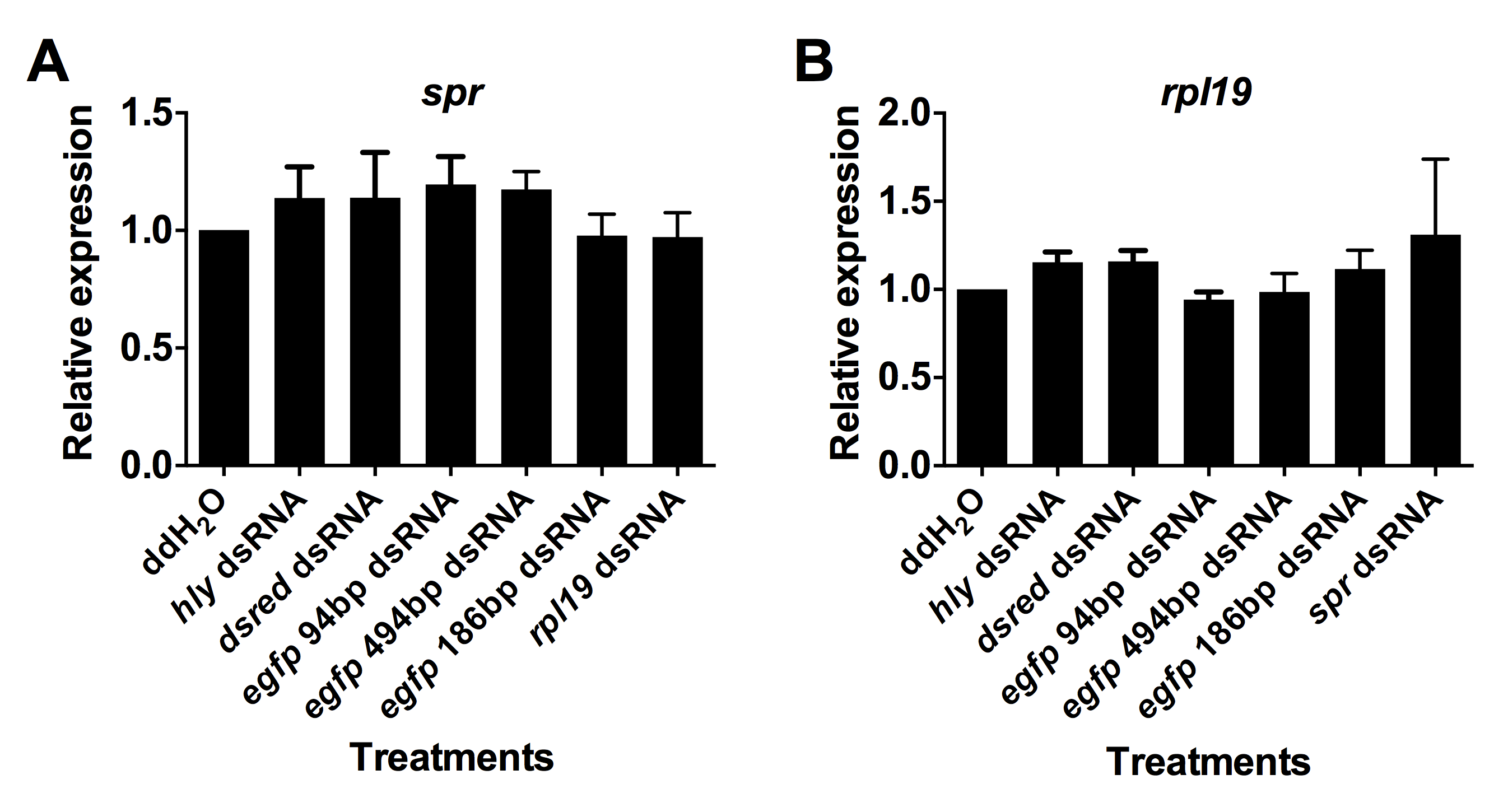
**

**Figure S2 Examination of possible off-target effects of dsRNAs.** Expression of spr and rpl19 in B. dorsalis after oral administration of dsRNAs and ddH_2_O, as determined using qRT-PCR. The normalised expression of the target gene is given relative to its expression in the ddH_2_O control, which was set to 1.

**
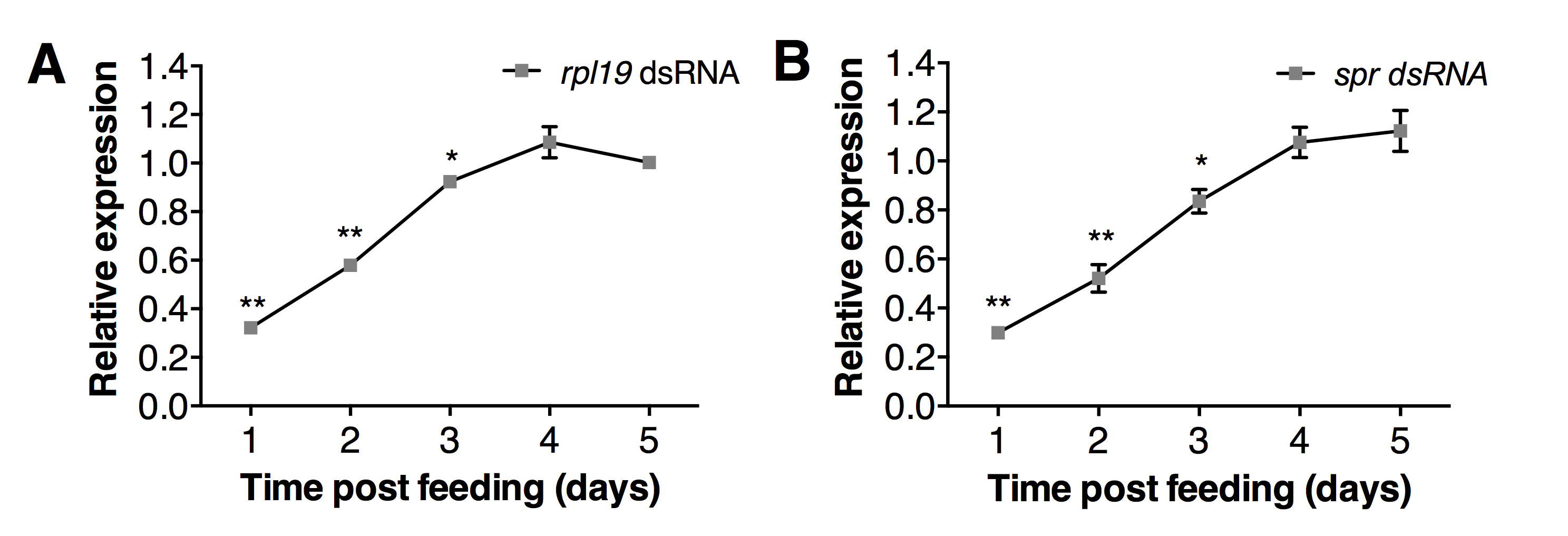
**

**Figure S3 rpl19 and *spr* expression pattern after** orally administered rpl19 dsRNA or spr dsRNA. *B.dorsalis* was fed 1000 ng/μl rpl19 dsRNA or *spr* dsRNA for 6 hr. The normalised expression of the target gene is given relative to its expression in the egfp dsRNA control, which was set to 1. All error bars represent the SE of the mean of three independent biological replicates. * and ** indicates a statistically significant difference in *rpl19* or *spr* expression compared with the control *egfp* dsRNA treatment (*, P<0.05, and **, P<0.01).

**
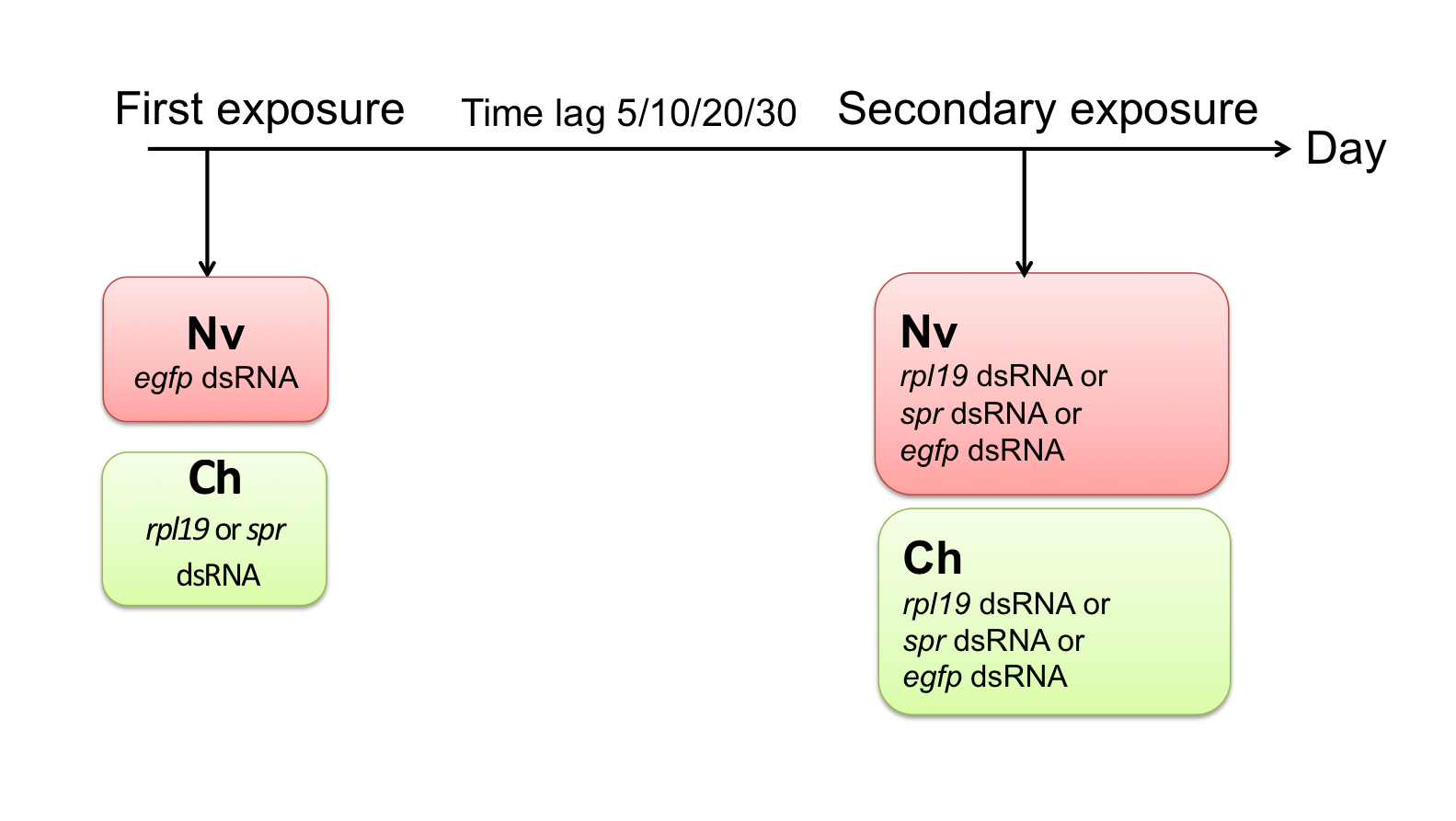
**

**Figure S4 Schematic representation of the experimental design.** The timeline depicts the experimental process in days. The flies were divided into two groups. The Nv group was fed egfp dsRNA in the first exposure, whereas the Ch group was fed either rpl19 dsRNA or spr dsRNA. We set the time lag between the first and second exposures to 5, 10, 20 or 30 days. For the secondary exposure, both the Nv and Ch groups were fed rpl19 dsRNA, spr dsRNA or control egfp dsRNA to induce RNAi.

**
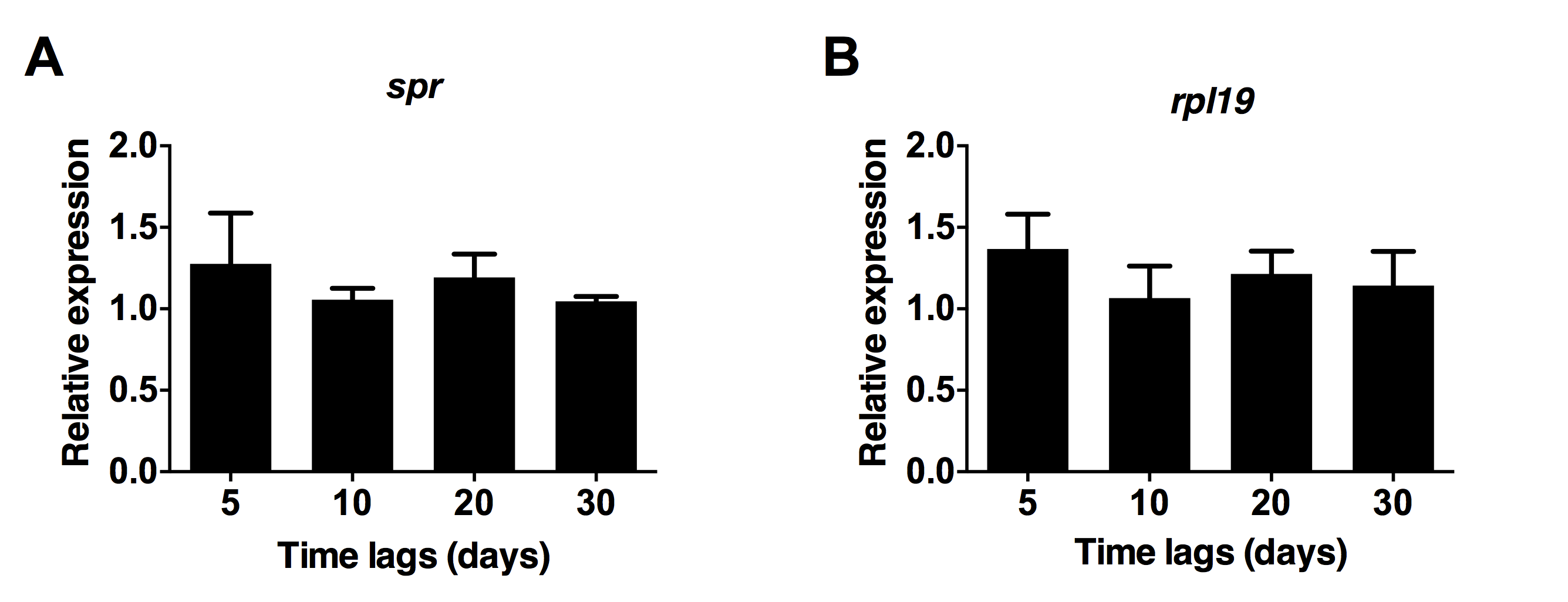
**

**Figure S5 The expression pattern of *spr* and *rpl19* in the untreated flies over time.** All error bars represent the SE of the mean of three independent biological replicates.

**
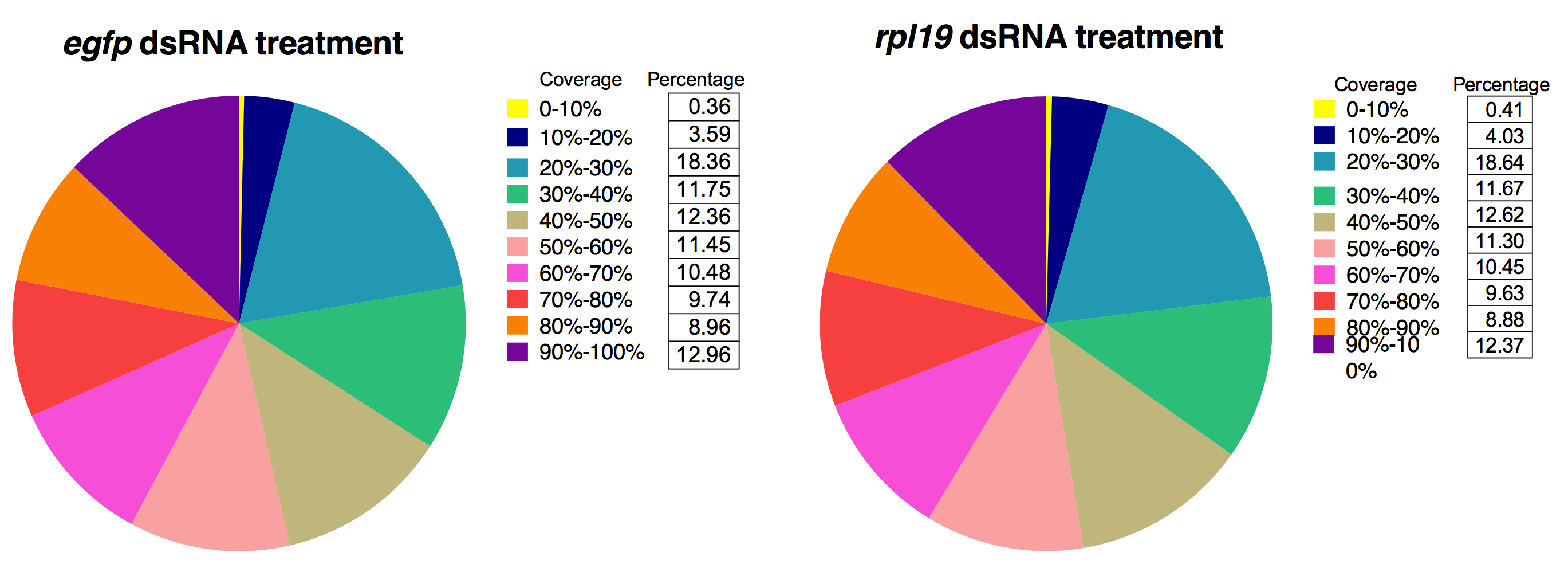
**

**Figure S6 Summary of mapping coverage to the unigenes in the transcriptome.**


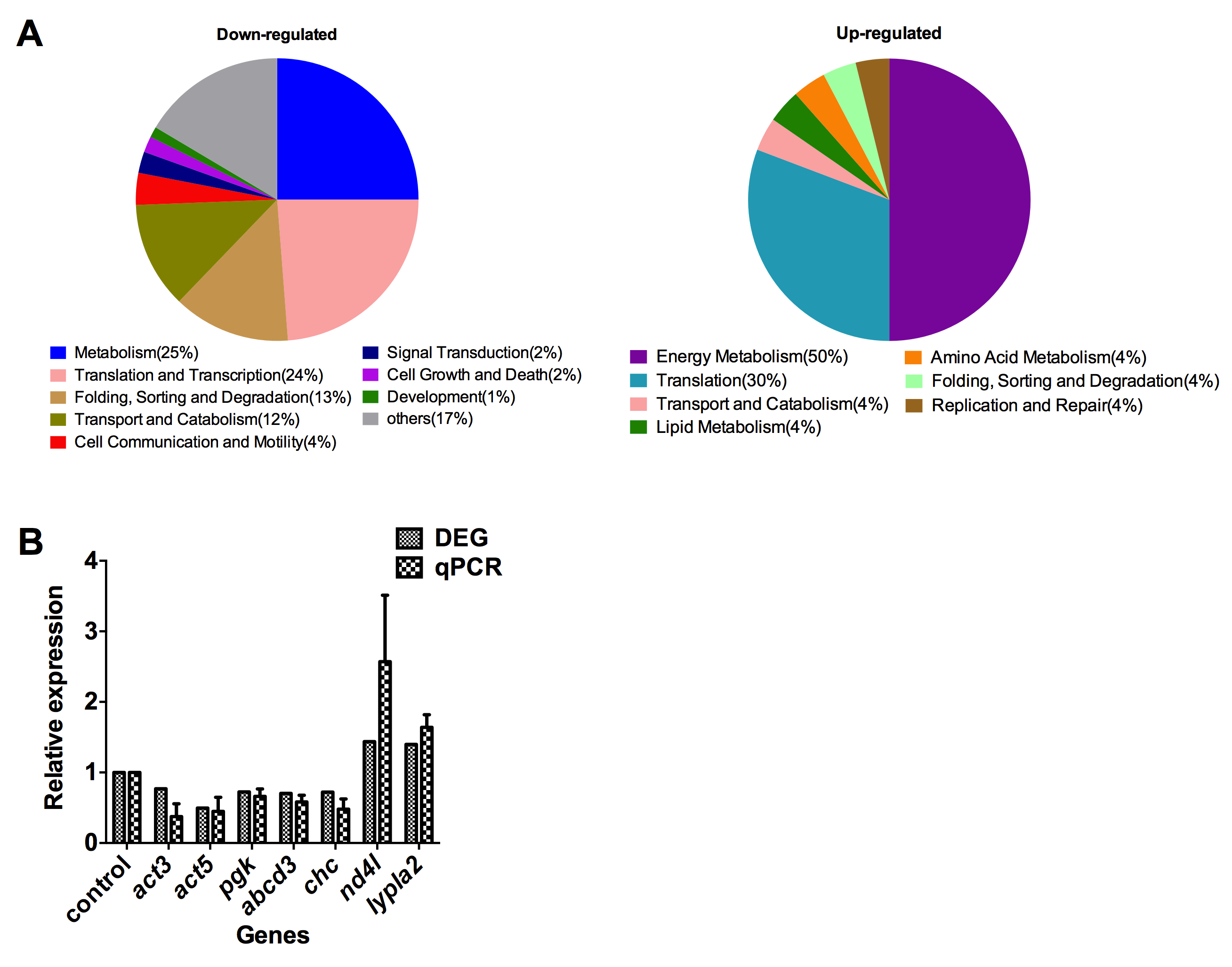


Figure S7 KEGG pathway analysis and validation of DGE sequencing. (a) Pie chart of up-regulated genes and down-regulated genes identified by KEGG pathway analysis. (b) qPCR validation of differentially expressed genes. The normalised expression of the target genes is given relative to their expression in the *egfp* dsRNA control, which was set to 1.

**TABLES**

Table S1 Summary of the DGE.

| Sample | *egfp* dsRNA treatment | *rpl19* dsRNA treatment |
| --- | --- | --- |
| Raw reads | 6512317 | 5973846 |
| Clean reads | 6363092（97.7%） | 5793444（96.9%） |
| Length | 100 | 100 |

Table S2 GO enrichment analysis of differentially expressed genes.

|  | GO term | P-value |
| --- | --- | --- |
| Cellular component | intracellular | 2.39E-33 |
|  | cytoplasm | 7.63E-32 |
|  | cell | 2.77E-24 |
|  | nucleic acid binding | 2.36E-07 |
|  | chromosome | 1.10E-05 |
| Molecular function | binding (other binding) | 5.90E-15 |
|  | biosynthesis | 2.14E-13 |
|  | electron transport | 2.17E-07 |
|  | membrane fusion | 1.99E-04 |
| Biological process | translation regulator activity | 1.17E-07 |
|  | development | 1.39E-07 |
|  | cell motility | 4.18E-05 |
|  | transport | 7.48E-05 |
|  | structural molecule activity | 8.89E-04 |

**Table S3 Oligonucleotide primers used in the manuscript.**

| Primer | Sequence(5’ to 3’ orientation) | Purpose |
| --- | --- | --- |
| Q*rpl19* F | GGCGGAGAAACAACGTAGCA | real-time qPCR |
| Q*rpl19* R | TGCAATTCTTTCCTCGCGGC | real-time qPCR |
| Q*sdha* F | CAAGTGCAAAGGTGTGTTGG | real-time qPCR |
| Q*sdha* R | GTCGCAATGACGGTGTTATG | real-time qPCR |
| Q*spr* F | AAAGCGCTCTTCCGCGATAA | real-time qPCR |
| Q*spr* R | ACCACAGCGATGGGTATTTC | real-time qPCR |
| Q*ap50* F | GCCACCGATACCACGTCCTT | real-time qPCR |
| Q*ap50* R | TCTGCTGATGAGTCCCCAGG | real-time qPCR |
| Q*gmer* F | CACTGGACTTGGCGAGACTA | real-time qPCR |
| Q*gmer* R | ATCAAATGCCTTCGCCACAG | real-time qPCR |
| Q*vha16-1* F | GCCGCCTATGGTACCGCTAA | real-time qPCR |
| Q*vha16-1* R | ACCAGCCATGACAACGGGAA | real-time qPCR |
| Q*idicp* F | ACCGGTGCTACAACTGCTCT | real-time qPCR |
| Q*idicp* R | GGCCAATTCTCCTGTCCATGC | real-time qPCR |
| Q*saposin* F | TCGGATTAGCAGCGCCCATT | real-time qPCR |
| Q*saposin* R | TCGTTCCCACCAGCCGAAAT | real-time qPCR |
| Q*vhasfd* F | TCACCGCATTCTGGGCATGT | real-time qPCR |
| Q*vhasfd* R | GGCATAGCCAGAAAAGCCAGCA | real-time qPCR |
| Q*arf72* F | TGTCTGACCACCCAAATCCCA | real-time qPCR |
| Q*arf72* R | TCTGCAAGTCGGTGAGGTGG | real-time qPCR |
| Q*pi3k* F | CAAGCCCTCCTCCATTGTTA | real-time qPCR |
| Q*pi3k* R | CGAGAGGCTGATAATGCACA | real-time qPCR |
| Q*rab7* F | CGTGGCGCTGATTGTTGTGT | real-time qPCR |
| Q*rab7* R | TCCGAGCACCACGAAAGGAA | real-time qPCR |
| Q*bet3* F | CAGATCGCATACAACAGGCC | real-time qPCR |
| Q*bet3* R | GAGGCAGTTCGACGAATTCC | real-time qPCR |
| Q*light* F | GGCCGATTACATTCCAAACAACG | real-time qPCR |
| Q*light* R | CGTCATGCCCGAGTCGTGAA | real-time qPCR |
| Q*ninaC* F | GAGCCCGGTGTATAACGAGA | real-time qPCR |
| Q*ninaC* R | ACGATCCCAATCCCAAACCT | real-time qPCR |
| Q*cog3* F | ACCAAACAGCTGCCGTTAAG | real-time qPCR |
| Q*cog3* R | TTTCACCCAGCAACTGTTCG | real-time qPCR |
| Q*chc* F | ACAGCAGGTCAATACGCCGA | real-time qPCR |
| Q*chc* R | AAGAGTGGTGGCGTCGTTGA | real-time qPCR |
| Q*act3* F | TTGCCCCACCAGAGCGTAAA | real-time qPCR |
| Q*act3* R | TTTGCGGTGGACAATGCCTG | real-time qPCR |
| Q*act5* F | CACCCGCAATGTATGTCGCC | real-time qPCR |
| Q*act5* R | GACACCATCGCCAGAGTCCA | real-time qPCR |
| Q*pgk* F | GTTCAAAGAGCCCGTATGCCG | real-time qPCR |
| Q*pgk* R | GCCACCTTCAGTAGCCGAGA | real-time qPCR |
| Q*abcd3* F | TGTTGTTATTGGTGGCCGCTTC | real-time qPCR |
| Primer | Sequence(5’ to 3’ orientation) | Purpose |
| Q*abcd3* R | TGAGCAGCGCGGATTTGAATTT | real-time qPCR |
| Q*nd4l* F | TTTAGAGTATGTGAAGGTGCTTTGG | real-time qPCR |
| Q*nd4l* R | AATCATTCCCATGTGTACGA | real-time qPCR |
| Q*lypla2* F | CAATGCCGGTGTCACTGAATGC | real-time qPCR |
| Q*lypla2* R | AGCACTTTTGATGCCCTCCTCA | real-time qPCR |
| *egfp* 186bp F | CGAGCTCACGTAAACGGCCACAAGTTC | plasmid construction |
| *egfp* 186bp R | CCCAAGCTTAAGTCGTGCTGCTTCATGTG | plasmid construction |
| *egfp* 92bp F | CGAGCTCGACGACGGCAACTACAAGAC | plasmid construction |
| *egfp* 92bp R | CCCAAGCTTTCCTTGAAGTCGATGCCCTT | plasmid construction |
| *egfp* 494bp F | CGAGCTCTAAACGGCCACAAGTTCAGC | plasmid construction |
| *egfp* 494bp R | CCCAAGCTTGGTGTTCTGCTGGTAGTGGT | plasmid construction |
| *hly* F | CGAGCTCACGGCCGTCAAGTTTATTTG | plasmid construction |
| *hly* R | CCCAAGCTTGTAAGTCTCCGAGGTTGCCA | plasmid construction |
| *rpl19* F | CGAGCTCGGTTTGGCTGGACCCTAATGA | plasmid construction |
| *rpl19* R | CCCAAGCTTGCATTCGAGCATTTGCAGTA | plasmid construction |
| *dsred* F | CGAGCTCGGGTAACTCACGGGGTATCC | plasmid construction |
| *dsred* R | CCCAAGCTTCTTTTCCGGACACAGTTCCG | plasmid construction |
| *spr* F | CGAGCTCAACAATCTACATCGCCCTCG | plasmid construction |
| *spr* R | CCCAAGCTTCGCTTTGCGTCGTTCCTT | plasmid construction |
